# Detergent Resistant Membrane Domains in Broccoli Plasma Membrane Associated to the Response to Salinity Stress

**DOI:** 10.3390/ijms21207694

**Published:** 2020-10-17

**Authors:** Lucía Yepes-Molina, Micaela Carvajal, Maria Carmen Martínez-Ballesta

**Affiliations:** 1 Group of Aquaporins, Department of Plant Nutrition, Centro de Edafología y Biología Aplicada del Segura (CEBAS-CSIC), Campus de Espinardo, E-30100 Murcia, Spain; lyepes@cebas.csic.es (L.Y.-M.); mcarvaja@cebas.csic.es (M.C.); 2 Department of Agronomy Engineering Universidad Politécnica de Cartagena, Paseo Alfonso XIII, 48, 30203 Cartagena (Murcia), Spain

**Keywords:** aquaporins, broccoli, detergent-resistant membrane, microdomains, plasma membrane, salt stress

## Abstract

Detergent-resistant membranes (DRMs) microdomains, or “raft lipids”, are key components of the plasma membrane (PM), being involved in membrane trafficking, signal transduction, cell wall metabolism or endocytosis. Proteins imbibed in these domains play important roles in these cellular functions, but there are few studies concerning DRMs under abiotic stress. In this work, we determine DRMs from the PM of broccoli roots, the lipid and protein content, the vesicles structure, their water osmotic permeability and a proteomic characterization focused mainly in aquaporin isoforms under salinity (80 mM NaCl). Based on biochemical lipid composition, higher fatty acid saturation and enriched sterol content under stress resulted in membranes, which decreased osmotic water permeability with regard to other PM vesicles, but this permeability was maintained under control and saline conditions; this maintenance may be related to a lower amount of total PIP1 and PIP2. Selective aquaporin isoforms related to the stress response such as PIP1;2 and PIP2;7 were found in DRMs and this protein partitioning may act as a mechanism to regulate aquaporins involved in the response to salt stress. Other proteins related to protein synthesis, metabolism and energy were identified in DRMs independently of the treatment, indicating their preference to organize in DMRs.

## 1. Introduction

Plant plasma membrane (PM) is a selective barrier between cells and the environment, playing a crucial role in the reception and transduction of environmental signals and the regulation of cell-environment communication [1]. Therefore, PM is important in essential functions such as cellular nutrition, endocytosis and response to environment stresses [2].

Salt stress is one of the most common abiotic stresses; it is widespread throughout the world and causes losses in the production of the most common crops. PM is important in the response and defence of plants against salinity because it is one of the cell parts that salt reaches first. Membrane lipids and transport proteins have an important function in regulating the permeability of PM, which triggers responses to salinity [3]. Since the control of water and nutrient distribution in the whole plant is an important factor in the acclimation of plants to a saline environment, the role of membrane proteins, such as aquaporins, is crucial, which has been widely reported in different species, such as *Arabidopsis* [4], maize [5] and broccoli [6]. Aquaporins work out mainly as channels to facilitate and regulate the permeability of water across cell membranes [7] and are representative markers of water uptake and whole plant water status [8]. Moreover, salinity modifies aquaporin expression and protein abundance, which indicates that these proteins must be involved in the tolerance response to salt stress [9].

Despite the multiple studies concerning PM, there are still many unknown and pending details to study, especially related to the distribution of proteins and lipids in the membrane. In this sense, several aspects with regard to membrane proteins and salinity stress response or other abiotic stresses cannot be elucidated with the classic “fluid mosaic” model for PM, which consists of a homogeneous lipid bilayer and randomly embedded proteins [10,11]. Regarding the PM structure, new approaches have appeared and enhanced recent models, revealing unknown aspects about the function of PM and the response to abiotic stress. The models consist of the existence of nanodomains (10–200 nm) [12], which are regions rich in sterols and sphingolipids [13]; this confers to PM a heterogeneous system. These domains are membrane-resident protein clusters in higher-order structures and are actually known as membrane nanodomains or “raft lipids” [14,15]. One of the most effective ways to determine specific functions of raft lipids in PM is through the isolation of detergent-resistant membranes (DRMs), that is, Triton X-100 (TX100) insoluble membrane fractions [16,17]. This methodology constitutes a biochemical approach in which the study of changes in DRMs in order to determine modification in the composition of raft domains in vivo was standard for a long time and is still a technique that is used today [18]. Moreover, it is possible to prepare membrane microdomains with detergent-free methods. These methods have certain advantages in that they involve fewer negative effects for lipid-lipid and protein-lipid interactions. On the other hand, detergent-free methods involve more contamination by other low-density membranes [19,20].

As it occurs in animal cells, proteomic analysis showed that DRMs in plants are involved in membrane trafficking, signal transduction, cell wall metabolism occurring on the PM and endocytosis [21,22,23]. Furthermore, numerous studies establish a relation between DRMs and a response to biotic stress [24] but the function of DRMs with regard to abiotic stress in plants is not entirely understood [25]. Only a few studies have been developed along these lines; for example, Minami et al. [17] showed alterations in proteomic profiles of DRMs during cold acclimation in *Arabidopsis* plants. However, there is evidence that elucidates an important relation between an abiotic stress response and proteins located in DRM fractions [18,25] such as aquaporins, heat shock proteins, actins and clathrins [2,22,25]. Li et al. [26] determined the partitioning in PM and trafficking of aquaporin PIP2:1 of *Arabidopsis* in controlled conditions and under salt stress (100 mM NaCl for two days). They showed that membrane rafts have an important role in this respect, since under salinity stress as well as clathrin-dependent endocytosis, membrane raft-associated endocytosis is involved.

Broccoli (*Brassica oleracea* L. var Italica) is a vegetable known to have a health-promoting effect, due to its high content of beneficial compounds, such as glucosinolates and isothiocyanates [27]; so this crop has an important economic and agronomic interest. Moreover, new ways to leverage broccoli byproducts are being investigated, for example, the use of vesicles from the plasma membrane of leaves and roots in biotechnological applications [28,29]. Interestingly, in a previous work, it was determined that these vesicles are more stable over time when they are obtained from plants that had been cultured under high salinity [29]. This stability is an important factor to consider when using these vesicles in biotechnological applications, such as for carriers of bioactive compounds in cosmetics, agriculture or pharmaceuticals [30,31].

Thus, the objective of this work was the investigation of the appearance of plasma membrane rafts through detergent-resistant membranes isolated from broccoli roots in order to determine lipids and protein characteristics. The changes in membrane rafts under saline stress were related to salinity tolerance. Different approaches were used, in general terms, to perform this characterization: Analysis of protein and lipid content, ultrastructural analysis and study of the size of the vesicles and measures of water-osmotic permeability. In addition, part of this study was focused on aquaporins, since these proteins play an important role in the response to saline stress. In this sense, western-blot analysis of PIP1 and PIP2 aquaporins and a proteomic analysis by means of LC−MS/MS were carried out in order to determine the distribution of different isoforms concerning detergent-resistant membranes.

## 2. Results

### 2.1. Quantitative Analysis of Protein Amounts in DRM Isolation

**Figure 1:**
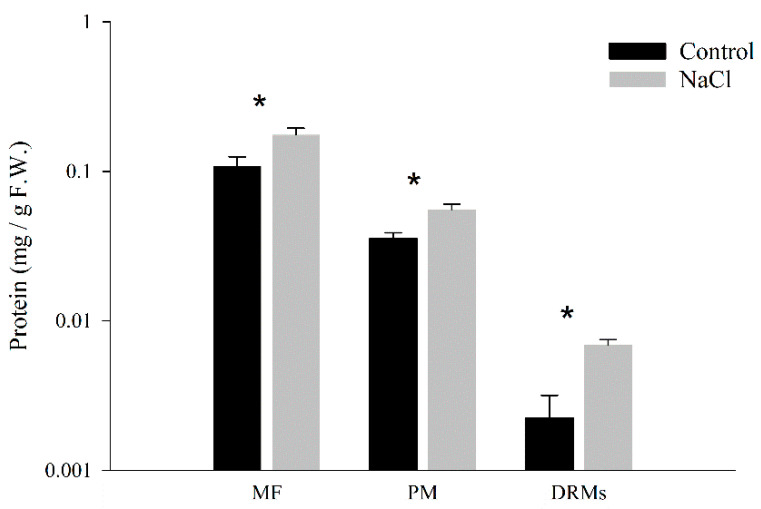
Protein concentration (mg g-1 FW) in microsomal fraction (MF), plasma membrane (PM) and detergent-resistant membranes (DRMs) from broccoli roots treated with 0 (control) and 80 mM NaCl. Data are means ± SE (*n* = 5). Analyses were done with three biological samples. Asterisks (*) represent significant differences according to *t*-test for each fraction (*p* < 0.05).

The protein amount in each membrane fraction from the microsomal fraction to the DRMs was determined and expressed by the weight of fresh tissue (Figure 1). The amount of protein was higher in membrane fractions obtained from NaCl plants. In control samples, the protein yield in DRMs was 5.56 ± 2.02% of that of the total PM and in NaCl samples this value was 12.51 ± 0.85%.

### 2.2. Lipid Composition of Broccoli Root DRMs Compared to PM

**Figure 2:**
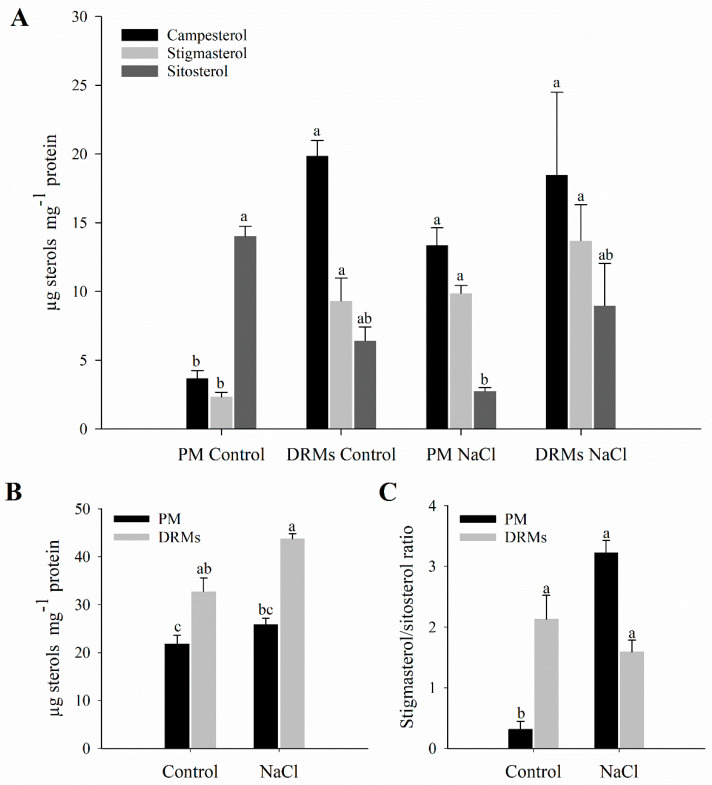
Sterol analysis. Campesterol, stigmasterol and sitosterol (µg mg^−1^ protein) (**A**), total sterols (µg mg^−1^ protein) (**B**) and stigmasterol/sitosterol ratio (**C**) in plasma membrane (PM) and detergent-resistant membranes (DRMs) isolated from broccoli roots control and treated with NaCl. Data are means ± SE (*n* = 3). Analyses were done with three biological samples. Columns with different letters (a, b, c) for each variable differ significantly according to Tukey’s test (*p* < 0.05).

The lipid composition of root PM and DRMs extracted from control and salt-treated plant was analysed. The sterol analyses of the PM and DRMs appear summarized in Figure 2. In PM fractions, exposure to NaCl (80 mM) for 15 days triggers a decrease in sitosterol content. On the contrary, regarding free sterols in DRMs, no statistically significant changes were observed comparing control and salinity. Furthermore, an increase in the content of campesterol in the DRM fraction compared to PM under control conditions was shown (Figure 2A). Regarding the total content of sterols, there is a statistically significant increase in DRMs with respect to PM in both control and NaCl conditions (Figure 2B). The ratio of sitosterol to stigmasterol increases in PM in salt-treated plants and does not change in DRMs if comparing control and NaCl conditions (Figure 2C).

On the other hand, in Table 1 fatty acid composition as a percentage is shown. This analysis revealed that in PM palmitoleic acid (C16:1) was the predominant fatty acid both in control and salt treated plants, but no significant differences appeared between treatments. At this salinity level there was a statistically significant increase in linoleic acid (C18:3). The unsaturation grade of the membrane fractions was determined by the RUFA (ratio of unsaturated fatty acids) and DBI (double bond index) indices. In this case, the RUFA increased significantly in PM isolated from roots of salt-treated plants relative to control plants. Regarding DRMs, palmitoleic acid (C16:1) is also the predominant fatty acid and an increase in oleic acid (C18:1) instead of in linoleic acid (C18:3) was produced under salinity. Besides, in DRMs, a decrease in RUFA was produced in salinity conditions. As for the differences between PM and DRM fractions, this analysis showed an increase in oleic acid (C18:1) in DRMs under salinity. In control, RUFA increased in DRM fractions, but under salinity this ratio decreased significantly in DRMs compared to PM.

### 2.3. Ultrastructural Analysis And Size of Broccoli Root DRMs Compared to PM

**Figure 3:**
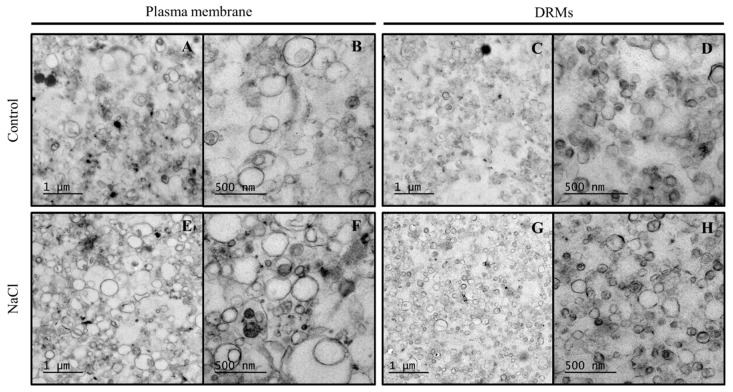
Transmission electronic microscopy images of control plasma membrane (PM) (1 µm) (**A**) and 500 nm (**B**), control detergent-resistant membranes (DRMs) (1 µm) (**C**) and 500 nm (**D**), NaCl PM (1 µm) (**E**) and 500 nm (**F**), and NaCl DRMs (1 µm) (**G**) and 500 nm (**H**).

Membrane fractions (PM and DRMs) were processed by chemical fixation. Electron microscopy images show that control PM (Figure 3A,B), NaCl PM (Figure 3E,F), control DRM (Figure 3C,D) and NaCl DRM (Figure 3G,H) fractions contained mostly membrane vesicles. It has to be noted that no change was observed between PM and DRMs obtained from control plants and plants grown under salinity conditions as far as structure is concerned.

**Figure 4:**
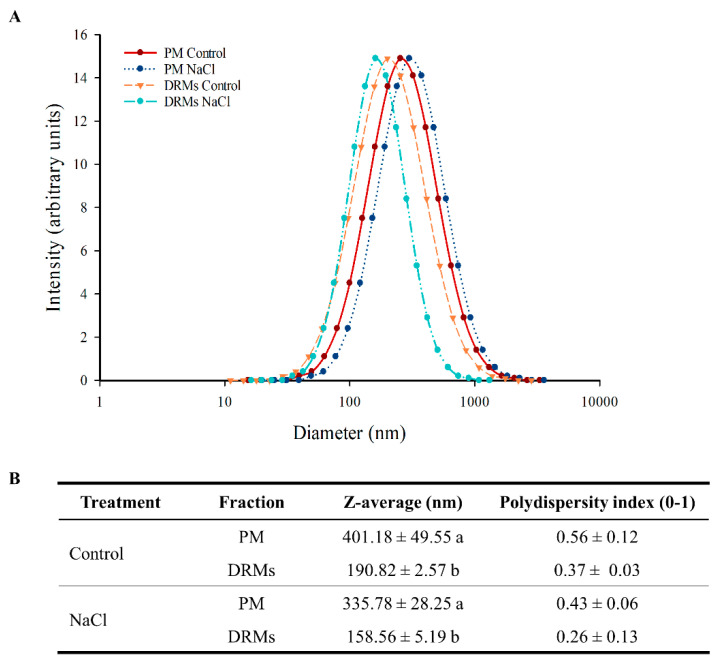
Size distribution (**A**) and average size and polydispersity index (**B**) of plasma membrane (PM) and detergent-resistant membranes (DRMs) from broccoli roots treated with 0 (control) and 80 mM NaCl. Data are means ± SE (*n* = 3). Analyses were done with three biological samples. Different letters (a, b) represent significant differences according to *t*-test for each fraction (*p* < 0.05).

The determinations of vesicle diameter appear in Figure 4 and reveal a significant difference between the PM and DRM vesicles. Dynamic light scattering of the PM and DRM vesicles under control and NaCl conditions showed the size distribution of the different vesicles (Figure 4A). PM had a larger diameter, but all populations are distributed in the range of 100 to 1000 nm (Figure 4A). Control PM vesicles had a diameter of 401.18 nm and NaCl PM vesicles 335.78 nm, but no significant statistical differences were found between the two populations of vesicles; similarly, there were no differences between control and NaCl DRM vesicles, but these vesicles had lower diameter than PM vesicles: In control conditions, DRMs vesicles were 210.38 nm smaller (190.82 nm) and under salt stress 177.22 nm (158.59 nm). Regarding the polydispersity index, in all samples the value ranged from 0.26 to 0.56 and no significant statistical differences were found (Figure 4B).

### 2.4. Vesicle Integrity and Functionality

**Figure 5:**
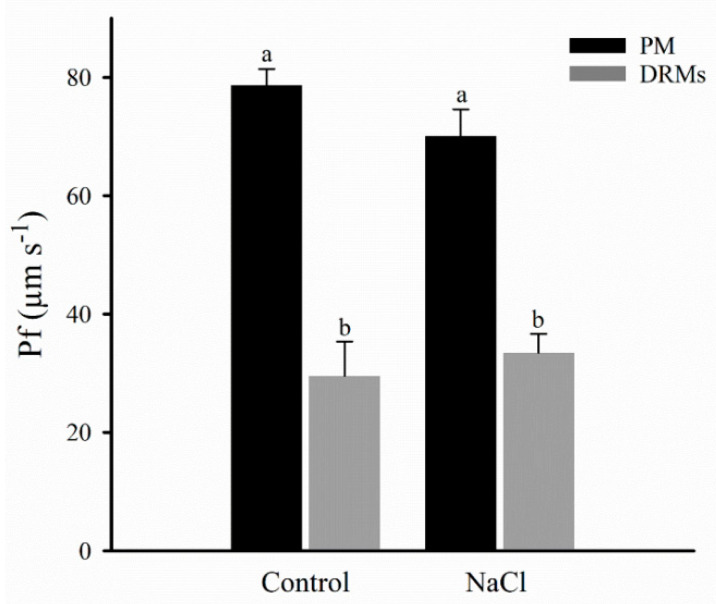
Osmotic water permeability, *Pf*, (µm s^−1^) of plasma membrane (PM) and detergent-resistant membranes (DRMs) from broccoli roots treated with 0 (control) and 80 mM NaCl. Data are means ± SE (*n* = 3). Analyses were done with three biological samples. Different letters represent significant differences according to two-way ANOVA and posterior Tukey-HSD test (*p* < 0.05).

The integrity and functionality of the PM and DRM vesicles were determined by stop-flow measurements by osmotic water permeability (*Pf*) measurement (Figure 5). The results show that the PM vesicles obtained from control broccoli roots had significantly higher *Pf* than DRM vesicles. However, *Pf* remained unchanged for the control and NaCl plants in both PM and DRMs.

### 2.5. PIP1 And PIP2 Aquaporin Quantification

**Figure 6:**
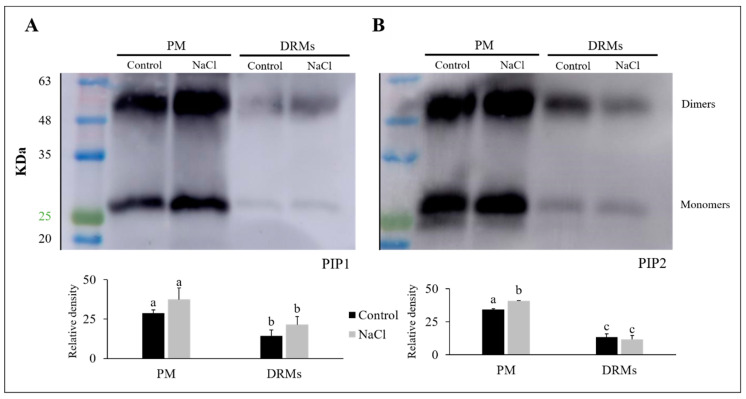
Immunodetection of PIP1 (**A**) and PIP2 (**B**) homologues in the root plasma membrane (PM) and detergent-resistant membranes (DRMs) of control broccoli plants and plants treated with 80 mM NaCl. Data are means ± SE (*n* = 3). Analyses were done with three biological samples. Different letters represent significant differences according to Tukey-HSD test (*p* < 0.05).

The abundance of PIP1 and PIP2 aquaporins was carried out using western blot analysis. Two bands corresponding to 29 KDa (aquaporin monomer) and 58 KDa (aquaporin dimer) were detected with PIP1 and PIP2 antibodies in PM and DRM (control and NaCl) (Figure 6). The immunostaining intensity differed among treatments and membrane fractions; this, moreover, depends on the PIP subfamily. The content of PIP1 increased both in PM and DRMs under saline stress, while a decrease in the total amount of PIP1 in DRM fractions with respect to PM was observed in control and NaCl (Figure 6A). Regarding the PIP2 subfamily (Figure 6B), we found that in PM there was an increase in the content of PIP2 when the broccoli was grown under saline stress, although this fact did not occur in DRMs, since in this fraction a decrease in PIP2 appeared under stress conditions. In DRM fractions there appeared a decrease in the content of PIP2 aquaporins, in the same way that occurred with the PIP1 subfamily.

### 2.6. Proteomic Analysis of DRMs from B. oleracea Root

**Figure 7:**
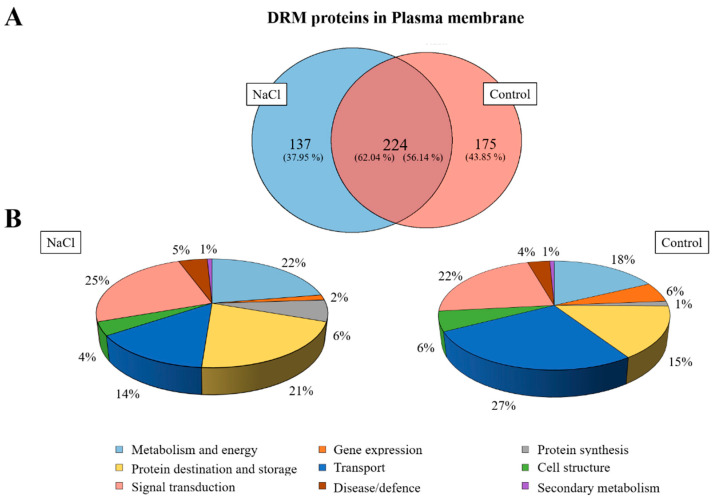
Venn diagram of control DRMs and NaCl DRM proteins identified in broccoli roots (**A**) and functional classification of identified proteins in both samples as percentages (**B**).

A Venn diagram was made according to the accession numbers of the identified protein in the proteomic analysis in both samples (DRM control and NaCl) after the specific selection of proteins was located in the PM in order to show proteins that were identified only in the control or in salt treatment in DRMs and proteins that were identified in both treatments (Figure 7A). The results obtained show that 224 proteins were shared between control and NaCl DRMs, and 175 and 137 proteins were categorized as unique to the control and NaCl DRMs, respectively.

Identified proteins were classified into nine functional categories according to [32]: Metabolism and energy, gene expression, protein synthesis, protein destination and storage, transport, cell structure, signal transduction, disease/defence and secondary metabolism. In Figure 7B, normalized distributions of functional categories in each sample are shown, and the results indicate that an increase in the proportion of proteins involved in transport and a decrease in proteins associated with protein destination and storage were detected in the saline treatment, although salt stress did not greatly alter the proportion of different types of proteins.

**Figure 8:**
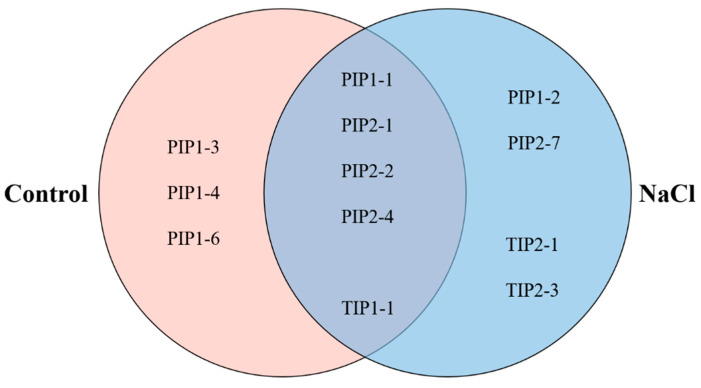
Venn diagram aquaporin distribution in detergent-resistant membranes (DRMs). Different sections show aquaporins found only in the control (**left**) and NaCl (**right**) and aquaporins present in both the control and NaCl (middle).

After a general proteomic analysis (Tables S1–S3), a focused search was carried out to localize all the aquaporins present in the DRMs both in the control and the NaCl samples (Table 2). In control DRMs, eight aquaporins belonging to two families (PIP and TIP) were located and, in NaCl DRMs, nine aquaporins of these two families appeared, too. In Figure 8, a distribution of the aquaporins found in both samples is represented. There are five aquaporins that appear in the DRM control and NaCl: PIP1-1, PIP2-1, PIP2-2, PIP2-4 and TIP1-1. On the other hand, there are some isoforms that have only been found in control or in NaCl. The aquaporin PIP1-3, PIP1-4 and PIP1-6 have only been detected in control DRMs. On the contrary, PIP1-2, PIP2-7, TIP2-1 and TIP2-3 have only appeared in DRMs isolated from salt-treated plants.

## 3. Discussion

One important modification in broccoli under salt stress was the chemical composition PM of both lipids [33] and proteins [29] that has been reported to relate transport and salt tolerance. In recent years, the role of proteins associated with lipid DRMs in plants has been the focus of many studies due to these domains being commonly reported as key systems in many biological processes, such as stress response [34]. Based on the idea that sterols are required for the formation of a liquid-ordered lipid phase in plants and that this fact will condition protein membrane location [35], in the present study we determined the DRMs isolated from the root PM of broccoli plants, describing the changes induced by salinity. Regarding the percentage of protein from the total PM recovery in DRMs, our results (5.56 2.02% in control and 12.51 0.85% in salinity) were in accordance with those obtained in previous reports in other plants as *Medicago truncatula* [23], *Arabidopsis thaliana* [17], *Avena sativa* and *Oryza sativa* [34], pointing out the general characteristics according to the protein/lipid ratio in DRMs. However, in our work, the protein level was higher in DRMs isolated from the PM of plants grown under NaCl than in the control. This result differed from the data obtained in *A. thaliana* plants under cold acclimation, in which the total protein recovered from DRMs was lower in plants grown under stress [17]. This could be due to the different stress tolerance response in *Arabidopsis*, very sensitive to abiotic stress [36], and *Brassica*, tolerant to abiotic stress [37], which could affect PM protein partitioning, resulting in a higher concentration of proteins in DRMs when plants are stress tolerant.

In addition to protein content, lipid composition of PM was previously reported to be largely affected by salinity [33,37]. Previously, the sterol analysis of PM from broccoli plants in control and salt conditions revealed results similar to this work. Thus, an increase of stigmasterol and a decrease of sitosterol were shown. It has been described that sitosterol plays a significant role in the ordering of fatty acid chains in the membrane, decreasing membrane water and ion permeability, and the activity of membrane proteins [38]. Thus, sitosterol decrease in broccoli PM roots could result in a mechanism to cope with salt stress. DRM lipid composition analysis evidenced an enrichment in total sterols with respect to PM in both, control and NaCl samples, which is an important characteristic that makes out lipid raft domains from the rest of the PM [13]. In other reports working with artificial proteoliposomes, cholesterol was found to induce higher resistance to detergent solubilization on a broader range of temperatures (from 4 to 15 °C) [39]. In our work, both control- and NaCl-treated DRMs differ mainly by increasing campesterol and decreasing sitosterol content. This has not been observed previously, but the campesterol has been related to membrane stabilization, tightly packing the phospholipid bilayer [40]. The authors described a strong ordering ability for campesterol, in the same range as the cholesterol one, and a less efficient sitosterol.

Moreover, our results showed an increase in oleic acid in DRMs under salt stress instead of linolenic acid as occurred in PM. Similarly, in the PM of safflower and broccoli plants an increase in linoleic acids under salt stress and a decrease in oleic acid have been observed [37]. However, fatty acid modifications in DRMs isolated from plant PM have not been previously described under abiotic stress conditions. In control conditions, however, the results are similar to PM except for an increase in linolenic acid increasing the unsaturation. An increase in oleic acid in NaCl-DRMs could reflect a membrane remodelling in order to maintain membrane environment integrity preservation after NaCl application. It has been observed that glycerolipids of tobacco plant DRMs contained more saturated fatty acyl chains, which would contribute to the rigidity of the liquid-ordered phase of membrane rafts [22]. However, this only occurs in our broccoli DRMs under salinity stress conditions, pointing to a stress response.

It has been reported that stability in DRM lipid content could be due to the fact that in these domains the lipids form a dense packing, increasing membrane viscosity to provide a conserved environment for proteins with specific biological functions [41]. Therefore, for raft functionality it has been stated that specific PM lipids, mainly sterols and fatty acids from phospholipids, could interact with proteins providing signalling and trafficking platforms in the PM [42]. From the results of this work, DRMs from broccoli plants are distinctive, but also the fact that NaCl plants provided higher levels of sterols and higher saturation of fatty acids could be related to the key for stress tolerance.

Transmission electron microscopy was used to characterize PM and DRM morphology from roots of broccoli plants. As has been already reported, isolated PM forms vesicular structures in vitro [43], which was also observed in both the control and NaCl PM vesicles from broccoli roots. Similarly, isolated DRMs from PM were likewise vesicular-shaped, although these vesicles were smaller. Vesicles of DRMs have also been described in DRMs isolated from mammals, such as from kidney cells [16], demyelinated membranes of rat brain [44] or human erythrocytes [45], and, in addition to vesicles, shaped membrane sheets may also appear in DRMs isolated from sucrose gradients. Moreover, in plant DRMs, similar morphology was observed and lined/shaped membrane fragments appeared together with vesicles [23]. Differences in the display of DRM ultrastructure could be due to the different detergent applied to PM, since detergent isolation may define membrane subdomains and specific lipid ensemble and structure [46].

The diameter of the vesicles was measured through light-scattering technique. PM vesicles in both control and NaCl, as we showed in several previous works [28,29], have a diameter between 300 and 400 nm. On the other hand, DRM vesicles were smaller than PM vesicles. In this case, the diameter of the control and NaCl DRM vesicles was less than 200 nm, which was in accordance with previous results [45], where DRM vesicles from human erythrocytes had a diameter around 100 nm. These types of vesicles could also be used as nanocarriers and, in addition, due to their smaller size, DRM vesicles could be suitable for a finer application, specifically as nanomedical devices [47]. The size of DRM vesicles obtained is difficult to relate with the size of the lipids raft domains in vivo because DRMs are not lipid raft in the same way that these domains are located in the plasma membrane in vivo [48]. The real size of the lipids raft in cells is still unclear and is still a matter of debate. Different studies reveal that these domains must be quite small; roughly, they set values between 50 and 200 nm [49].

The osmotic water permeability coefficient (*Pf*) was measured in PM and DRM vesicles using stopped-flow light scattering. In DRMs isolated from the PM of broccoli roots, this measure determined the vesicles’ integrity and functionality, which is of interest for their potential biotechnological use. Our results reported similar *Pf* in vesicles isolated from broccoli plants grown under salt stress regarding the control, in both PM and DRM membrane fractions. As occurred for PM, where salt stress induced changes in lipid composition without altering water transport properties [33], a similar effect was described in DRMs from broccoli plants.

Moreover, *Pf* was lower in DRM vesicles than in PM vesicles; this could be due to the smaller aquaporin content in DRMs compared with PM vesicles, as was shown in the western analysis. However, it has been postulated that water permeability of AQP4 depends on the bilayer composition [50] and this fact must also be further considered in our DMRs, as well as differences in aquaporin isoform partitioning in DRMs and PM [26]. Belugin et al. [51] observed that osmotic water conductivity was significantly lower in the bilayers containing raft components. The authors proposed that, in DMRs, aquaporins had a high affinity to sterol molecules, being in close contact with them. This may cause changes in the water permeability of the protein, leading to the channel “closure” form upon such contact or to an “opening” state when this contact is disrupted. Similarly, the conductivity of *Nt*AQP1 for CO_2_ and of *Nt*PIP2;1 for water resulted in a lower sterol environment [52].

The amount of PIP1 and PIP2 present in the different types of vesicles were determined by SDS-PAGE electrophoresis and western blots. A base-containing Triton X-114 kit was used for membrane protein extraction, followed by a urea/NaOH clean-up of proteins [53,54,55] in order to remove peripheral proteins and concentrate membrane proteins. However, while Triton X-114 phase partitioning has been previously used for vesicle membrane protein isolation, demonstrating that the detergent phase fraction contained more transmembrane proteins than other fractions [56], difficulties for complete transmembrane protein isolation cannot be ruled out, but the protein yield and purity are in accordance with our previous work [57].

Regarding PM vesicles, similar results to others already published [33,58,59] were observed; that is, PIP1 aquaporins scarcely changed due to saline stress but PIP2 aquaporins increased significantly under this stress condition. Aquaporins belonging to the PIP subfamily have been described as playing an important role in salt stress, since this stress involves both water and ionic stress. This increase in the amount of PIP2 under conditions of excess salt has been proposed as a cellular mechanism of roots to promote the transport of water within the cells and thus dilute NaCl to avoid the toxicity caused by excess NaCl in the whole plant [58]. Thereby, aquaporins and specially PIP2 aquaporins, should be a type of stress-response protein [58]. As far as PIP aquaporins in DRMs are concerned, some studies carried out in four-week-old *Arabidopsis* seedlings and seven-day etiolated pea (*Pisum sativum* L.) seedlings have shown that in DRMs isolated from PM there was a higher amount of these proteins than in PM fractions [17,60]. However, this did not occur in our work, where a lower amount of PIP1 and PIP2 isoforms were found in DRMs compared to PM, in both control and salt conditions. These differences regarding previous results, in addition to the genotype, could be due to the fact that DMR isolations were carried out from the roots of one-month broccoli plants and at this stage plant physiology differs from that of the seedling stage. In any case, in our broccoli plants, PIP1 and PIP2 isoforms were positioned in DRMs in a greater proportion under salinity and control conditions, respectively. While partitioning of PIP2;1 was associated with membrane rafts after 10 min of 100 mM NaCl exposition in a PM and DRM-dynamic partitioning [26], the long-term effect of salinity seems to operate in a different way in broccoli plants when compared to control and NaCl DRMs. However, western analysis did not consider all PIP1 and PIP2 isoforms and there are few works in which the aquaporins in DRMs have been investigated under different stress conditions. Further research to explain in detail the presence of aquaporins in DRMs and PM at the isoform level is needed.

A proteomic study was performed in order to characterize the proteomic profile in DRMs isolated from control and NaCl roots of broccoli plants. In previous works, proteomic analyses have been realised to find differences between the proteomic profile in PM and DRMs [1] or to characterise the proteome of DRMs in different plants [17,22,23], but there is no information about protein identification in DRMs isolated from the root of salt-treated broccoli plants compared to control DRMs. In this proteomic study, some possible contaminating proteins were identified. Soluble proteins are highly abundant and could appear as contaminates, but others such as ribosomal proteins have been identified in these samples due to cytoskeletal-bound polysomes [61,62]. In addition, a search in the Uniprot database was carried out against a very broad taxonomic group in order to provide more extensive protein identification, but discrepancies and overlaps may have appeared when the same protein appeared in several organisms.

In our study, a similar protein distribution by categories was found in control and NaCl DRMs. In both cases, we found that the majority of proteins present in DRMs were proteins involved in destination and storage, metabolism and energy, transport and signal transduction. In these categories, some proteins have been reported to be associated and to play an important role in DRMs such as H^+^-ATPase, aquaporin, tubulin and clathrin [2,17]. Pang et al. [63] showed in a proteomic study of *Arabidopsis* and *Thellungiella spp* plants that these proteins involved in energy, metabolism and protein synthesis play an important role in the response to salinity. Under low salt conditions (50 mM) *Arabidopsis* plants enhanced their energy metabolism to deal with the stress due to water and osmotic homeostasis perturbation [64], where plants have to regulate different energetically dependent processes. In the same study, some proteins with a role in the protein synthesis in *Arabidopsis* were up-regulated under salt stress, such as the elongation factor 1-β-alpha-subunit 2 protein, which is necessary to ensure the normal process of protein synthesis [63]. This fact was also revealed when the overexpression of the sugar beet translation initiation factor improved salt tolerance and protein synthesis under salt stress in *Arabidopsis* [65].

It is widely known that aquaporins belonging to the PIP subfamily were associated with DRMs and several isoforms have been identified in these domains under control conditions in leaves from *Nicotiana tabacum* cv. Xanthi (PIP1-5), *N. tabacum* BY-2 cells (PIP1-5, PIP2-1 and PIP2-4) [2,22], cotyledons from *A. thaliana* (PIP1-2, PIP1-3, PIP1-5, PIP2-1, PIP2-6 and PIP2-8) [66], *A. thaliana* callus (PIP1-2 and PIP2-8) [67] and *A. thaliana* seedlings (PIP1-2, PIP1-4, PIP1-5, PIP2-1, PIP2-2, PIP2-3, PIP2-5, PIP2-6 and PIP2-7) [17].

In our study PIP1-5, PIP2-3, PIP2-5, PIP2-6 and PIP2-8 have not been detected in DRMs, but other PIP isoforms that had not been previously described were identified, such as PIP1-1 and PIP1-6. In addition to the PIPs, three TIPs (TIP1-1, TIP2-1 and TIP2-3) were identified in DRMs. The presence of TIP aquaporins in DRMs has not been not widely described, although Krügel et al. [68] showed a TIP aquaporin associated to the DMR fraction from potato leaves. Under our analysis conditions, PIP1-2 and PIP2-7 were only detected in DRMs from NaCl-treated plants, these isoforms have been associated to a possible role in saline stress. In other studies, carried out under saline stress, increased *PIP1-2* and *PIP2-7* expression was observed, which reveals a role of these aquaporins in the response to salt stress. The authors suggest a possible function in the maintenance of water uptake and in the balance in the plant [69]. Similarly and also in *Arabidopsis* plants, it has been suggested that saline stress triggers a removal of PIP2-7 from the PM [70] and this fact could be carried out through raft domain-dependent endocytosis, although this assumption requires further research. Thus, these two isoforms are in higher amounts in DRMs allowing their identification in broccoli plants and NaCl may participate as a signal to its reorganization in DRMs. DRMs have been proposed as sterol- and sphingolipid-enriched domains, where specific proteins are associated for their translocation [41]; in our plants, PIP1-2 and PIP2-7 were aquaporin isoforms partitioned into the PM.

Moreover, there were two TIPs (TIP2-1 and TIP2-3) that were only detected in DRMs isolated from a plant under salt stress. In a similar way as occurs with PIP2-7, TIP2-1 could be recruited in raft domains, since it seems that this protein increases sensitivity to saline stress and its membrane location may be important for enhancing stress tolerance [71]. Whether the ability to temporally organize individual aquaporin isoforms, while excluding others in DRMs, is related to a distinct aquaporin cycling rate from/to the PM or different aquaporin sensibility to salinity with implications in the stress response, needs further investigation.

## 4. Materials and Methods

### 4.1. Plant Growth

Seeds of broccoli (*Brassica oleracea* L. var. Italica cv. Parthenon) were germinated and cultivated as previously reported in [9] with some modifications. The pre-hydration with deionized water in continuous aeration was carried out for 24 h. The seeds were germinated in vermiculite in the dark at 28 °C for two days and the sprouts were then transferred and cultivated in hydroponic solution in a controlled-environment chamber. After two weeks of growth, a saline treatment (80 mM NaCl) was applied to half of the plants. The roots from control and treatment plants were harvested for plasma membrane and detergent-resistant membrane isolation after another two weeks of growth.

### 4.2. Plasma Membrane Isolation and Enzyme Assay

A pooling of samples with five different plants of each treatment was used for 10 membrane extractions in order to get large enough amounts of proteins for analysis. Root PMs were purified from microsomal fractions using the two-phase aqueous polymer technique first described by [72] and modified by [57]. Roots were cut before vacuum filtering with 0.5 g of PVP and 160 mL of extraction buffer (0.5 M sucrose, 1 mM DTT, 50 mM HEPES and 1.37 mM ascorbic acid, pH 7.5). The samples were homogenized using a blender and filtered through a nylon mesh (pore diameter of 100 µm). The homogenate was centrifuged at 10,000× *g* for 30 min at 4 °C. The supernatant was centrifuged at 100,000× *g* for 35 min at 4 °C. Then, the pellet was suspended in a FAB buffer (5 mM PBS and 0.25 sucrose, pH 6.5). Afterwards, 2 mL of microsomal fractions were introduced into the two-phase system (PEG-3350/Dextran-T500 6.3% (*w*/*w*)) in the presence of 5 mM KCl, 330 mM sucrose, 2.5 mM NaF and 5 mM potassium phosphate pH 7.8. The system was centrifuged at 4000× *g* for 5 min and the upper phase was diluted with a solution containing 9 mM KCl, 0.2 M EDTA, 0.2 M EGTA, 0.5 M NaF and 10 mM Tris-borate, pH 8.3. The mixture was centrifuged at 100,000× *g* for 35 min. The final pellet containing the PM fraction was suspended in the FAB buffer.

The purity of the PM was estimated after measuring the enzymatic activity characteristics of the plasma membrane and other organelles (Table S4). The activity of the PM-associated, vanadate-sensitive ATPase was assayed [73]. The activities of nitrate sensitive ATPase [74], latent inosine diphosphatase [75] and cytochrome C oxidase [76] were used as enzymatic markers of tonoplast, Golgi apparatus and mitochondria, respectively. Enzymatic activities were determined in a thermostated Thermo-Spectronic spectrophotometer at 25 °C. The final extracted PMs were washed in a buffer containing 2 mM EDTA, 2 mM EGTA, 100 mM NaCl, 5 mM Tris-HCl, pH 8, and centrifuged at 100,000× *g* for 30 min and finally resuspended in 9 mM KCl, 300 mM sucrose, 5 mM Na_2_EDTA, 5 mM Na_2_EGTA, 50 mM NaF, 5 mM dithiothreitol, 2 µg/mL leupeptin, 10 mM Tris-borate, pH 8.3.

### 4.3. Detergent-Resistant Membrane (DRM) Isolation

Plasma membranes were resuspended in a Tris-buffered saline (TBS) buffer (140 mM NaCl, 3 mM KCl, 25 mM Tris-HCl, pH 7.5) and treated with 1% Triton X-100 (*w*/*v*) for 30 min on ice with shaking every 10 min as previously described [2] and [23]. Solubilized membranes were placed at the ultracentrifuge tube and mixed with 60% sucrose in TBS buffer (*w*/*w*) to reach a concentration of 48% and overlaid carefully with successive 3 mL steps of 40%, 35% and 30% sucrose in TBS buffer (*w*/*w*) to generate a gradient. Then, the sucrose gradient was centrifuged for 20 h at 100,000× *g* in a SW28 rotor (Beckman). After centrifugation, DRMs were recovered at 30–35% interface (opaque band) and detergent-soluble fractions (DSF) were recovered at the bottom. DRMs and DSF were diluted in TBS buffer, and centrifuged for 4 h at 100,000× *g*. DRMs obtained by sucrose gradient were used for total protein quantification, lipid analysis, transmission electron microscopy (TEM), size of membranes and stopped flow light scattering.

### 4.4. Isolation of Proteins Associated with Membrane Rafts and Plasma Membrane

Proteins associated with membrane rafts and plasma membrane were isolated from control and salt-treated (80 mM) broccoli roots by two different kits from Bio-Rad (Bio-Rad Laboratories, Inc., Hercules, CA, USA): (1) The ReadyPrep™ Protein Extraction Kit-Signal for proteins associated with raft lipid, which employs differential solubilization at 4 °C in the presence of Triton X-100 to isolate proteins associated with membrane rafts and (2) the ReadyPrep™ Protein Extraction Kit-Membrane I for proteins associated with membranes, both following the recommendations of the manufacturer. The samples obtained by the protein extraction kits were used for western-blot and proteomic analyses.

### 4.5. Total Protein Quantification

The protein concentrations from microsomal fraction (MF), plasma membrane (PM) and detergent-resistant membranes (DRMs) were determined with an RC DC Protein Assay kit (BioRad), using BSA as standard.

### 4.6. Lipid Analysis

Sterol and fatty acids were determined as described by [77]. A mixture of chloroform-methanol (1:2, 0.75 mL) was added in an Eppendorf tube to different membrane fractions obtained with method I (0.5 mL), along with β-cholestanol (20 µL, 0.1 mg mL^−1^) used here as an internal standard for sterol analysis. Chloroform (CHCl3; 0.25 mL) was added and the mixture was shaken and centrifuged at 10,000× *g* for 6 min. The CHCl3 layer was retained, evaporated to dryness under N2 and made up to 100 µL with CHCl3. For sterol analysis, 20 µL of the CHCl3 extract was placed in a glass vial (2 mL), evaporated to dryness under N2 and acetylated using pyridine (50 µL) and Ac2O (100 µL). After 2 h, the solvents were evaporated under N2, ethyl acetate (20 µL) was added and the sterol analysed by GC using an HP5-bonded capillary column (30 m–0.25 mm–0.25 lm) coupled to a flame ionisation detector (FID), with H2 as carrier (1 mL min^−1^) and a temperature programme of 120–260 °C at 5 °C min^−1^, then 260–280 °C at 2 °C min^−1^ and finally 280–300 °C at 6 °C min^−1^. The injector and detector temperatures were 150 and 320 °C, respectively. Bound fatty acids were determined by using 20-ll portions of the CHCl3 extract; evaporating them to dryness under N2, transmethylating with sodium methoxide (0.5 N) in methanol (0.5 mL) and heating at 30 °C for 7 min. The resultant fatty acids methyl esters were extracted with hexane (1 mL), evaporated under N2, dissolved in ethyl acetate (20 µL) and analysed by GC using an HP5-bonded capillary column (30 m × 0.25 mm × 0.25 µm), with FID, He as carrier (1 mL min^−1^) and a temperature programme of 150–195 °C at 3 °C min^−1^, then 195–220 °C at 2 °C min^−1^ and finally 220–300 °C at 6 °C min^−1^. The injector and detector temperatures were 280 and 300 °C, respectively.

### 4.7. Transmission Electron Microscopy

PM and DRMs from broccoli roots were pelleted at 100,000 g. For chemical fixation, pelleted vesicles were sequentially fixed with glutaraldehyde (2.5% in 100 mM phosphate buffer, 2 h at 4 °C), osmium tetroxide (1% buffered, 2 h at 4 °C), and tannic acid (1% in deionized water, 30 min at 22 °C). The pellets were then thoroughly rinsed with water and covered with 2% low melting point agarose, then dehydrated with ethanol and epoxypropane at 22 °C and embedded in Epon. Blocks were sectioned on a Leica EM UC6 ultramicrotome, collected on Formvar-coated copper grids and stained with uranyl acetate followed by lead citrate. Sections were examined using a JEOL 1011 transmission electron microscope with digital camera GATAN ORIUS SC200. For each treatment, an average of 5–10 ultrathin sections were examined.

### 4.8. Size of Membranes Vesicles

The average size of the membranes vesicles, PM and DRMs, was checked using light-scattering technology; through intensity measurements with a Malvern ZetaSizer Nano XL machine (Malvern Instruments Ltd., Orsay, France), as previously described in [78]. This allowed the analysis of particles with a size range from 1 nm to 3 μm.

### 4.9. Stopped-Flow Light Scattering

The osmotic water permeability (*Pf*) was measured by the velocity of the volume adjustment of the membrane vesicles after changing the osmotic potential of the surrounding media. The volume of the vesicles (PM and DRMs) was followed by 90° light scattering at λex = 515 nm. Measurements were carried out at 20 °C in a PiStar-180 Spectrometer (Applied Photophysics, Leatherhead, UK), as described previously [79].

### 4.10. Gel Electrophoresis and Immunoblotting

Protein (10 μg per lane) was loaded for 12% sodium dodecyl sulfate-polyacrylamide gel electrophoresis (SDS-PAGE) [9]. The antibodies used were against the 42 N-terminous residues of PIP1:1 from A. thaliana (kindly provided by Prof. Dr Schäffner) and against a 17-amino-acid C-terminal peptide of A. thaliana PIP2:2 (kindly provided by Dr. Santoni). The first antibody (PIP1;1) used in broccoli plants recognise four different PIP1 (PIP1;1, PIP1;2, PIP1;3 and PIP1;4) and the second antibody (PIP2;2) recognise different PIP2 aquaporins isoforms. Goat anti-rabbit IgG coupled to horseradish peroxidase was used as the secondary antibody. A chemiluminescent signal was developed using the West-Pico Super Signal substrate (Pierce, Rockford, IL, USA). The intensity of each band was determined by ImageJ software [80].

### 4.11. Proteomic Analysis

#### 4.11.1. In-Gel Protein Digestion (Stacking Gel)

Each sample was diluted with loading sample buffer and then applied onto 1.2-cm wide wells of a conventional SDS-PAGE gel (1mm-thick, 4% stacking, and 12% resolving) (Figure S1). Then run was stopped as soon as the front entered 1 cm into the resolving gel, so that the whole proteome became concentrated in the stacking/resolving gel interface. The unseparated protein bands were visualized by Coomassie staining, excised, cut into cubes (1 mm^2^), deposited in 96-well plates and processed automatically in a Proteineer DP (Bruker Daltonics, Bremen, Germany). The digestion protocol used was based on [81] with minor variations: gel plugs were washed firstly with 50 mM ammonium bicarbonate and secondly with ACN prior to reduction with 10 mM DTT in 25 mM ammonium bicarbonate solution, and alkylation was carried out with 55 mM IAA in 50 mM ammonium bicarbonate solution. Gel pieces were then rinsed firstly with 50 mM ammonium bicarbonate and secondly with ACN, and then were dried under a stream of nitrogen. Proteomics Grade Trypsin (Sigma Aldrich) at a final concentration of 16 ng/μL in 25% ACN/50 mM ammonium bicarbonate solution was added and the digestion took place at 37 °C for 4h. The reaction was stopped by adding 50%ACN/0.5%TFA for peptide extraction. The tryptic eluted peptides were dried by speed-vacuum centrifugation.

#### 4.11.2. Liquid Chromatography and Mass Spectrometer Analysis (LC-ESI-MS/MS)

A 1 µg aliquot of each digested sample was subjected to 1D-nano LC ESI-MSMS analysis using a nano-liquid chromatography system (Eksigent Technologies nanoLC Ultra 1D plus, SCIEX, Foster City, CA, USA) coupled to a high speed Triple TOF 5600 mass spectrometer (SCIEX, Foster City, CA, USA) with a Nanospray III source. The analytical column used was a silica-based reversed phase Acquity UPLC^®^ M-Class Peptide BEH C18 Column, 75 µm × 150 mm, 1.7 µm particle size and 130 Å pore size (Waters). The trap column was a C18 Acclaim PepMapTM 100 (Thermo Scientific), 100 µm × 2 cm, 5 µm particle diameter, 100 Å pore size, switched on-line with the analytical column. The loading pump delivered a solution of 0.1% formic acid in water at 2 µL/min. The nano-pump provided a flow-rate of 250 nL/min and was operated under gradient elution conditions. Peptides were separated using a 150 min gradient ranging from 2% to 90% mobile phase B (mobile phase A: 2% acetonitrile, 0.1% formic acid; mobile phase B: 100% acetonitrile, 0.1% formic acid). The injection volume was 5 µL.

Data acquisition was performed with a TripleTOF 5600 System (SCIEX, Foster City, CA, USA). Data was acquired using an ionspray voltage floating (ISVF) 2300 V, curtain gas (CUR) 35, interface heater temperature (IHT) 150, ion source gas 1 (GS1) 25, declustering potential (DP) 100 V. All data were acquired using the information-dependent acquisition (IDA) mode with Analyst TF 1.7 software (SCIEX, USA). For IDA parameters, a 0.25 s MS survey scan in the mass range of 350–1250 Da was followed by 35 MS/MS scans of 100 ms in the mass range of 100–1800 (total cycle time: 4 s). Switching criteria were set to ions greater than mass to charge ratio (*m*/*z*) 350 and smaller than *m*/*z* 1250 with a charge state of 2–5 and an abundance threshold of more than 90 counts (cps). Former target ions were excluded for 15 s. The IDA rolling collision energy (CE) parameter script was used for automatically controlling the CE.

#### 4.11.3. Proteomics Data Analysis and Sequence Search

Mass spectrometry data obtained were processed using PeakView v2.2 Software (SCIEX) and exported as mgf files which were found using Mascot Server v2.6.0 (Matrix Science, London, UK) against the Brassicaceae protein database from Uniprot (last update: 20181026, 483.295 sequences), together with commonly occurring contaminants. Search parameters were set as follows: Enzyme, trypsin; allowed missed cleavages, 2; carbamidomethyl (C) as fixed modification and acetyl (Protein N-term), pyrrolidone from E, pyrrolidone from Q and oxidation (M) as variable modifications. Peptide mass tolerance was set to ±25 ppm for precursors and 0.05 Da for fragment masses. The confidence interval for protein identification was set to ≥95% (*p* < 0.05) and only peptides with an individual ion score above 20 were considered correctly identified. Predictions of transmembrane helices (TMH) (Table S5) were performed with TMHMM Server v. 2.0 [82], available at http://www.cbs.dtu.dk/services/TMHMM/. Predictions of protein location were performed with DeepLoc-1.0 [83] and UniProtKB [84].

### 4.12. Data Analysis

The statistical analyses were carried out using R software [85]. *t*-Test and Tukey’s HSD test and *p* < 0.05 was chosen to determine significant differences between treatments. Small letters or asterisks point to the significant differences between different samples.

## 5. Conclusions

In summary, as the biophysical and chemical characterization is fundamental for studying lipid/protein interactions in order to better understand the molecular relationships, the basis of domain segregation, dynamics, signaling and function are important features. In our results, the fact that the chemical composition of these domains was related to stress response in broccoli plants should be considered. In DRMs, typical high content of sterols appeared. However, salinity stress under our salt stress conditions increased fatty acid saturation, providing a higher liquid-ordered phase that turned out to be relevant for protein localization. This attribute could be related with stress tolerance that induced changes in lipid composition without altering membrane water transport properties regarding control. A minor but selective aquaporin content in sterol enriched domains compared with the rest of PM indicated that specific isoforms involved in salt stress response such as PIP1;2 and PIP2;7 preferred an enriched sterol or liquid-ordered environment. DRMs broccoli proteome indicated increased protein content with the main category, transport, and decreased protein destination and storage. Whether there is a protein-protein interaction between proteins or a narrow relation with aquaporin isoforms needs further investigation, but in any case a clear partitioning of these proteins into the membrane was associated with the broccoli response to salt stress. Regarding the next studies focusing on DRMs and protein partitioning, different efforts must be focused on the mechanisms that control the spatial and temporal plasma membrane lateral subdomains and protein remobilization under salt stress.

## Figures and Tables

**Table 1:** The fatty acid percentage (%), double bond index (DBI = (unsaturated fatty acid × number of double bonds)) and ratio of unsaturated fatty acids (RUFA = (18:2 + 18:3/18:1)) in the plasma membrane (PM) and detergent-resistant membranes (DRMs) of roots from control and salt-treated broccoli plants.

Fraction	Treatment	% Fatty Acids					
		Palmitoleic (C16:1)	Oleic (C18:1)	Linoleic (C18:2)	Linolenic (C18:3)	DBI	RUFA
Control	PM	56.14 ± 8.18 a	10.99 ± 0.04 ab	29.02 ± 1.64 a	1.96 ± 0.36 b	129.40 ± 2.96 a	1.77 ± 0.50 b
	DRMs	68.62 ± 10.46 a	5.23 ± 1.92 b	14.61 ± 4.46 ab	11.53 ± 4.08 a	136.28 ± 13.61 a	5.01 ± 0.14 a
NaCl	PM	60.91 ± 6.43 a	7.91 ± 3.24 b	20.46 ± 5.54 a	11.87 ± 2.67 a	137.66 ± 2.22 a	4.94 ± 1.27 a
	DRMs	53.62 ± 10.07 a	34.77 ± 9.57 a	5.77 ± 0.30 b	7.21 ± 1.13 a	117.4 ± 0.70 a	0.38 ± 0.09 c

Data are means ± SE (*n* = 3). Analyses were done with three biological samples. Values followed by different letters (a, b, c) in each column differ significantly according to Tukey’s test (*p* < 0.05).

**Table 2:** Aquaporins identified in the control and NaCl detergent-resistant membranes (DRMs) after a database search. MW is molecular weight (in dalton), PSMs is the number of fragmentation spectra acquired, peptides is the number of distinct peptide sequences in the protein group and coverage is the percentage of the protein sequence covered by identified peptides.

UniProt Accession	Description	MW (Da)	Score	PSMs	Peptides	Coverage
**Control**						
A0A178VIZ0	PIP1-1	30,897	72	2	1	21
A0A078IDF7	PIP1-1	30,852	206	4	3	29.7
A0A0D3C6T1	PIP1-3	30,825	135	2	2	29.7
A0A0D3DZM3	PIP1-4	30,769	194	2	2	29.7
F2X5K3	PIP1-6	16,451	198	2	2	21.3
A0A0D3B5C8	PIP2-1	30,208	736	18	9	39.6
O80369	PIP2-1	30,670	380	8	6	39.7
A0A0D3BRA6	PIP2-2	30,512	137	3	2	39.3
A0A0D3BRA5	PIP2-2	30,495	108	2	2	39.3
A0A0D3B2E7	PIP2-4	30,293	466	7	6	37.9
A0A078F7W0	PIP2-4	27,697	151	2	2	35.1
A0A0D3C4M6	TIP1-1	25,635	59	3	2	6.8
A0A078JDN1	TIP2-2	25,148	106	2	2	7.2
**NaCl**						
A0A178VIZ0	PIP1-1	30,897	61	2	1	21
R0HDM0	PIP1-1	30,779	273	4	4	29
A0A078G0R2	PIP1-2	30,792	961	19	13	35
A0A078HJQ7	PIP1-2	30,736	177	2	2	35
A0A0D3BRA6	PIP2-1	30,512	74	2	1	34
O80369	PIP2-1	30,670	293	6	4	29
M4CLX8	PIP2-2	25,497	59	2	2	27
A0A0D3B2E7	PIP2-4	30,293	472	7	7	38
A0A078IUF9	PIP2-7	29,949	91	3	2	30.2
A0A0D3C4M6	TIP1-1	25,635	130	3	3	7
A0A078GXB0	TIP2-1	24,983	205	3	3	7.3
A0A078J7S3	TIP2-3	25,348	165	6	2	7.2

## Supplementary Materials

The following are available online at https://www.mdpi.com/1422-0067/21/20/7694/s1, Figure S1: 12% SDS-PAGE stained with Coomassie Brilliant Blue of purified DRMs proteins isolated from roots of control and NaCl-treated broccoli plants. Table S1: Proteins identified in detergent resistant membranes (DRMs) isolated from control broccoli roots, after database search. Where MW is molecular weight (in dalton), PSMs is the number of fragmentation spectra acquired, peptides; the number of distinct peptide sequences in the protein group, emPAI is Exponentially Modified Protein Abundance Index and coverage; the percentage of the protein sequence covered by identified peptides. Table S2: Proteins identified in detergent resistant membranes (DRMs) isolated from NaCl (80 mM) broccoli roots, after database search. Where MW is molecular weight (in dalton), PSMs is the number of fragmentation spectra acquired, peptides; the number of distinct peptide sequences in the protein group, emPAI is Exponentially Modified Protein Abundance Index and coverage; the percentage of the protein sequence covered by identified peptides. Table S3: Proteins identified in detergent resistant membranes (DRMs) isolated from control and NaCl (80 mM) broccoli roots, after database search. Where MW is molecular weight (in dalton), PSMs is the number of fragmentation spectra acquired, peptides; the number of distinct peptide sequences in the protein group, emPAI is Exponentially Modified Protein Abundance Index and coverage; the percentage of the protein sequence covered by identified peptides. Table S4: Enzymatic activities (nmol min^−1^ mg^−1^ Protein) of control PM and microsomal fractions measured in the supernatant of the purification after aqueous polymer two-phase partitioning method.
